# High spatial resolution dataset of grapevine yield components at the within-field level

**DOI:** 10.1016/j.dib.2023.109580

**Published:** 2023-09-15

**Authors:** Baptiste Oger, Yulin Zhang, Jean-Philippe Gras, Yoann Valloo, Pauline Faure, Guilhem Brunel, Bruno Tisseyre

**Affiliations:** ITAP, Univ. Montpellier, INRAE, Institut Agro, 2 Place Pierre Viala, 34060 Montpellier, France

**Keywords:** Grapes, Missing vines, Dead vines, Spatial data, Vegetation index, Soil resistivity

## Abstract

In order to enhance the understanding of vine yield development and facilitate the design of innovative agricultural practices in viticulture (i.e., new estimation methods), it is essential to have accurate and detailed data on vine yield components, including unproductive vines, number of bunches, and bunch weight. However, obtaining accurate and high spatial resolution yield data at the vine scale is costly and difficult to have for the main yield components (number of bunches, weight of bunch, missing plants, etc.). As a result, existing vine yield data are frequently estimated or measured at the field level. Unfortunately, the accuracy of these vine yield data is insufficient to study the intricate relationships between different yield components and their spatial distribution within vineyards. In this context, this article proposes a complete vine yield dataset that was specifically collected to develop and to test new sampling protocols in precision viticulture.

This dataset comprises a comprehensive mapping of vine yield at the plant scale over two vine fields located in the southern region of France. Both vine fields were planted with the *Vitis vinifera*: cv. Syrah. The first field (Field 1) occupies 0.8 ha and data were collected in 2022, while the second field (Field 2) has an area of 0.5 ha and data were collected in 2008. Throughout the growing season, information regarding unproductive vines, inflorescence number, and bunch weight was collected for both vine fields. For both fields, at the flowering stage, the location of each productive and unproductive vines (dead and missing vines) was georeferenced, and the number of inflorescences was manually counted for all productive vines. For Field 1, at harvest, all bunches of the field were manually weighed with an accuracy of ±1 gram and georeferenced precisely (one point per vine). For each vine, total yield (grams per vine) was then computed as as the sum of the weight of its bunches. For Field 2, at harvest, the total yield per vine was estimated based on the weighing of representative bunches obtained from several regularly spaced set of 5 vines. In addition to the yield data, two ancillary data, including soil apparent resistivity measurements and common vegetative index derived from remote sensed imagery, are provided for both vine fields. Overall, the dataset consists of 3644 vines, with 2151 being productive, along with a total count of 33354 inflorescences and 19635 manually weighed bunches at harvest.

This dataset is of interest as it contains information on grape yield organization at the within-field level. This dataset could be used to assess the impact of unproductive vines on neighbouring vines yield, as well as the correlations between available ancillary data and all yield components.

Specifications Table

**Table:** 

Subject	Agricultural Sciences: Agronomy and Crop Science
Specific subject area	Characterization of vineyard yields and all its components (flowers, missing plants, bunch weight) at the within-field scale
Type of data	Georeferenced point data Table
How the data were acquired	Yield component data were acquired by manual counting and bunches weighing on two fields located in South of France. Vegetation index and soil apparent resistivity were respectively obtained with multispectral remote sensing imagery and soil conductivity sensors. Data were georeferenced using a high precision GNSS RTK (Global Navigation Satellite System Real-Time Kinematic) for the first vine field and DGPS (Differential Global Positioning System) for the second vine field.
Data format	Raw Aggregated
Description of data collection	The different yield components (number of productive vines, number of bunches per productive vine and bunch mass) were measured and georeferenced on two fields. These latter were chosen to be representative of southern France and of reasonable size. Additional available ancillary data (Vegetation index and soil conductivity) are also provided.
Data source location	• Institution: ○ Field 1: Institut Agro Montpellier ○ Field 2: INRAE Pech Rouge • City/Town/Region: ○ Field 1: Villeneuve-lès-Maguelone ○ Field 2: Gruissan • Country: France • Latitude and longitude: ○ Field 1: 43.547417; 3.8414769 ○ Field 2: 43.144561; 3.1310519
Data accessibility	Repository name: Zenodo Data identification number: 10.5281/zenodo.8318944 Direct URL to data: https://zenodo.org/record/8318944
Related research article	[1] Oger, B., Laurent, C. Vismara, P. & Tisseyre B, 2023. How to best estimate bunch number at vineyard level? OENO One, *57(3), 27–39.*https://doi.org/10.20870/oeno-one.2023.57.3.7404

## 1 Value of the Data

• The data can be useful to any work aiming at studying the impact of missing, dead and unproductive vines on each yield components (e.g., number of inflorescences/bunches, weight of bunches) and yield in general (Sanmartin et al. 2017 [1]).
• These data can be used to investigate new sampling approaches based on high resolution ancillary data like soil apparent resistivity or vegetation index to improve yield estimation before harvest (Acevedo-Opazo et al. 2008 [2], Carrillo et al. 2016 [3]).
• The dataset provided may also constitute data to study spatial distribution of grape yield at the within field level as well as any interaction between yield components (in addition to soil and plant vigour) at this scale. (Laurent et al. 2021 [4], Taylor et al. 2004 [5]).

## 2 Objective

Data were collected as part of several research projects on the characterization and estimation of vineyard yields at the field level. The initial objective of this dataset was to study how yield components were spatially distributed and how they could be better estimated with new sampling approaches. One research paper based on this dataset has already been published (Oger et al., 2023 [6]). That article focuses on estimation of the number of inflorescences through sampling. Bunch weight data, vegetation and soil resistivity have not yet been included in any scientific publication but they will soon.

Another article in the same scientific journal by Gras et al [7] presents additional yield data obtained from the same domain as Field 1. These data come from a different experimental setup, have lower spatial resolution but are available for the entire domain. These two articles provide an opportunity to explore complementary questions in viticulture. If relevant, meteorological data collected on the domain can also be retrieved from the article by Gras et al. [7].

## 3 Data Description

This dataset is composed of two types of data: raw data and filtered data. Raw data are simple georeferenced observations provided in shapefile format. Filtered data summarize available yield information derived from raw data. They are provided in a csv (Comma-Separated Values) file format.

### 3.1 Raw data

**Fig. 1:**
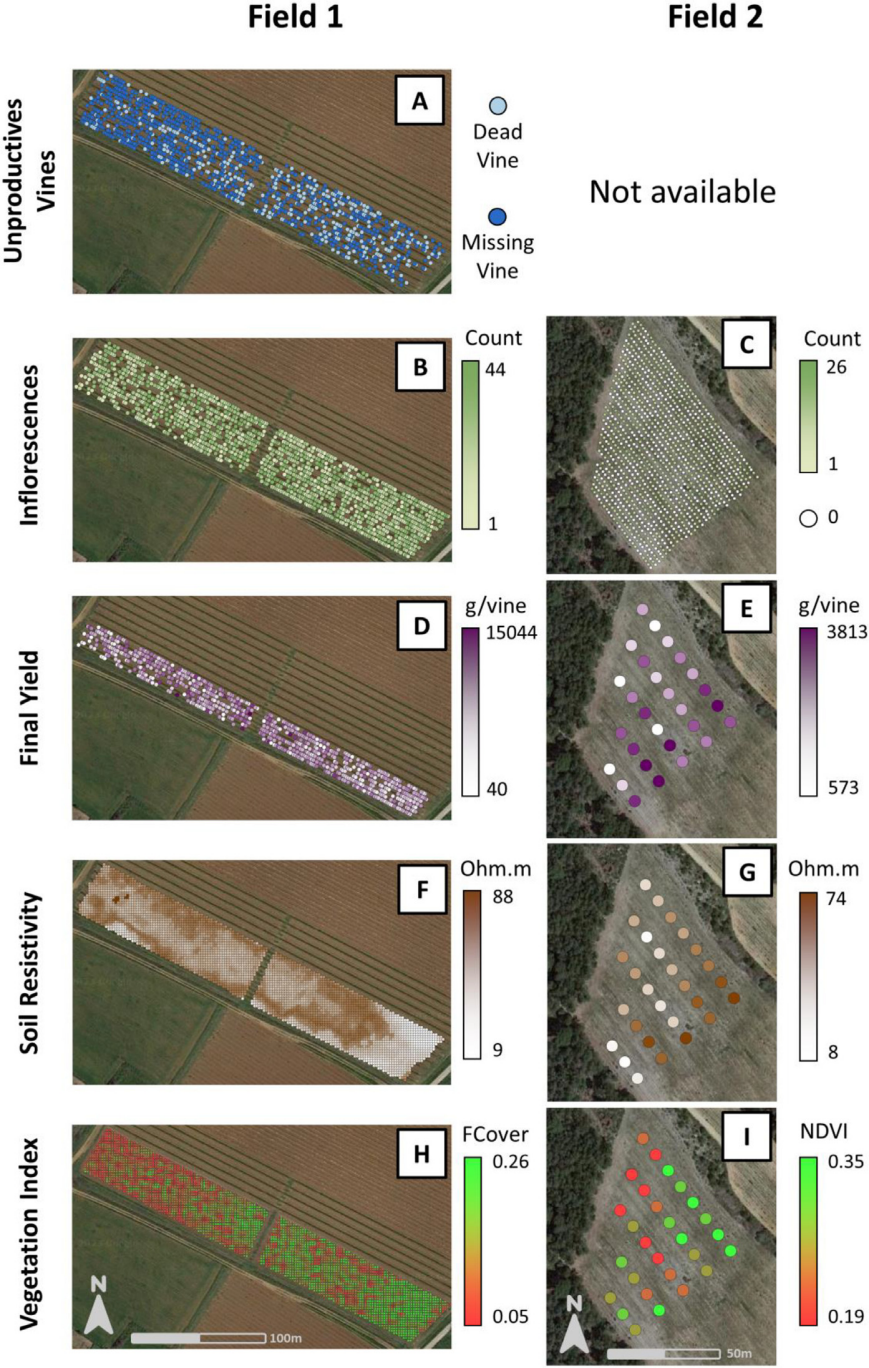
Maps of raw data for unproductive vine (A), inflorescence counting (B & C), final yield information (D & E), soil resistivity (F & G) and vegetation indexes (H & I) respectively for Field 1 and Field 2.

Raw data includes 9 shapefiles (.shp), one per data type and per field. Fig. 1 shows maps of these nine data files.

• “Field1_Dead_Missing_Vines.shp” (Fig. 1.A) contains the location of missing and dead vine identified in Field 1;
• “Field1_Inflorescences.shp” and “Field2_Inflorescences.shp” (Fig. 1.B and C) both contain the location and the number of inflorescences per vine counted during flowering;
• “Field1_Final_Yield.shp” and “Field2_ Final_Yield.shp” (Fig. 1.D and E) both contain location and measured values of yield weight per vine at harvest. For Field 1, the list of the bunch weight is also available;
• “Field1_Soil_Resistivity.shp” and “Field2_ Soil_Resistivity.shp” (Fig. 1.F and G) contain the electrical resistivity measurements of the soil on each field;
• “Field1_Vegetation_Index.shp” and “Field2_ Vegetation_Index.shp” (Fig. 1.H and I) contain vegetation index values, NDVI (Normalized Difference Vegetation Index, without unit) for Field 1 and FCover (Fraction of vegetation Cover, in %) for Field 2.

### 3.2 Aggregated data

Aggregated data are composed of three csv files, two for Field 1 and one for Field 2. “Field1_Yield.csv” and “Field2_Yield.csv” aggregate all available yield data for each vine plant (either productive or other types). In both these csv files, each line represents a planted vine. They are 2614 planted vines for Field 1 and 1030 planted vines for Field 2. Table 1 summarizes how spatial coordinates and yield parameters are organised within these two files.

**Table 1:** Description of the data files “Field1_Yield.csv” and “Field2_Yield.csv”.

Field Name	Description
ID	A unique identifier for each planted vine (number)
Row	The row identifier on which the vine is planted
X	X coordinates (EPSG : 2154 - RGF93 v1 / Lambert-93 – France)
Y	Y coordinates (EPSG : 2154 - RGF93 v1 / Lambert-93 – France)
Type	The vine status: can be “productive”, “unproductive”, “dead” or “missing” for Field 1 and “productive” or “unproductive” for Field 2
Inflorescence_number	The number of inflorescences per vine counted at flowering period
Total_yield	The total weight of grapes harvested from each vine. (g/vine). Only available for Field 1
Bunch_list	A list containing the weight of each bunch. Values are separated by semicolons. Only available for Field 1

Another file named “Field1_Bunches.csv” contains another representation of the data for Field 1. In this file, each row corresponds to a weighed bunch. The file was directly generated from “Field1_Yield.csv,” and the script used is provided with the data. Table 2 summarizes this third file.

**Table 2:** Description of the data file “Field1_Bunches.csv”.

Field Name	Description
ID	The identifier of the vine that bears the bunch
Row	The row or position of the vine that bears the bunch
X	X coordinates (EPSG : 2154 - RGF93 v1 / Lambert-93 – France)
Y	Y coordinates (EPSG : 2154 - RGF93 v1 / Lambert-93 – France)
Inflorescence_number	The number of inflorescences counted during the flowering of the vine
Total_yield	The total weight of grapes harvested from the vine that bears the bunch (g/vine)
Bunch_weight	The weight of the bunch (g)

## 4 Experimental Design, Materials and Methods

**Fig. 2:**
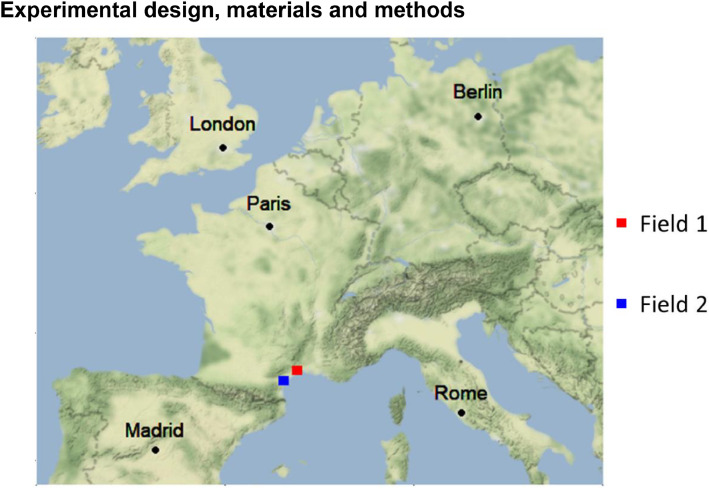
Location of the two fields in southern France.

The data come from two vine fields in production in the South of France (Fig. 2). Their properties are described in Table 3.

**Table 3:** Properties of the vine fields.

	Variety	Rootstock	Inter-plant distance (m)	Inter-row-distance (m)	Area (ha)
Field 1	Syrah	Sélection Oppenheim n°4	1.2	2.5	0.8
Field 2	Syrah	Ruggeri 140	1	2.5	0.5

Data acquisition campaigns were carried out in different contexts and in two different years, respectively 2022 and 2008 for Field 1 and Field 2. As a result, data acquisition protocols were not exactly the same for the two vine fields. All coordinates are expressed according to the French system (Datum RGF93, projection Lambert93 - EPSG: 2154).

### 4.1 Unproductive, dead and missing vines

In this section, a missing vine refers to a vine that has been uprooted, a dead vine refers to a vine that no longer has leaves, and an unproductive vine refers to a vine that does not produce bunches but has not been classified as missing or dead.

Missing and dead vines were only georeferenced on Field 1. A single operator made all observations manually. The location of missing and dead vines was recorded via the Mergin Map application (Lutra Consulting, UK) on a smartphone connected to a GNSS RTK (Global Navigation Satellite System Real-Time Kinematic) receiver and connected to the Centipede RTK network (Ancelin et al. 2022 [8]) to reach a centimetric accuracy. The acquisition of this data took place in early May of the year 2022.

For Field 2, missing plants were taken into account when counting bunches, and were considered as the location where 0 bunches were observed (see “Inflorescences” bellow).

### 4.2 Inflorescences

The number of inflorescences per vine was manually counted. Counting operations for inflorescences were conducted exhaustively for each vine on both fields.

In Field 1, the location of the vines was recorded using the Mergin Map application (Lutra Consulting, UK) on a smartphone connected to a GNSS RTK (Real-Time Kinematic) system on May 10th, 2022. Vines without inflorescences, including dead, missing, and unproductive vines, were disregarded during the counting process.

In Field 2, all vines were taken into account. Dead, missing, and unproductive vines were recorded as vines with zero inflorescence. As a result, there is no distinction between missing vine, dead vines and unproductive vines in this field. The counting was conducted in 2008, the vines (including missing vines) position were determined using a DGPS (Differential Global Positioning System) (Leica Geosystems company, model GS 50 with differential correction OMNISTAR) and the plantation density of the vine fields (10 cm accuracy).

### 4.3 Bunch weight and final yield

For Field 1, yield measurements were conducted exhaustively on half of the field (7 rows out of 14) on September 9, 2022. All vines with bunches were precisely located using a GNSS RTK and the Mergin Maps application (Lutra Consulting, UK). The bunches were weighed manually using scales with a precision of ± 1 g. For a given vine, the weight of each bunch was recorded in a form designed through the Mergin Maps application. 2022 was a particularly hot year in France, which led to the development of many smaller bunches after a second flowering. As a result, the number of bunches observed on the vines was really high and often exceeded fifty or even a hundred. Smallest bunches, weighting less than 30 g, were omitted as they did not contribute significantly to the total yield. For each vine, the total yield was calculated as the sum of the weight of its bunches.

In the case of Field 2, the final evaluation of yield was conducted only on specific sampling sites. These sampling sites consisted of five consecutive vines in a row, and their locations were determined based on a regular grid pattern of 15 m × 9 m, ensuring even distribution (Fig. 1.E). At each sampling site, the number of bunches was counted, and ten representative bunches were selected for weighing. The total yield was then calculated by multiplying the average weight of the ten bunches by the number of bunches.

### 4.4 Soil resistivity

For Field 1, soil resistivity measurements were conducted with a service provider company Geocarta system (Geocarta, France) on the whole field. The measurements were taken with electrode Wiener devices giving a maximal depth of investigation of 1 m (Panissod et al. 1997 [9]).

For Field 2, measurements of soil electrical resistivity were performed with a Wiener 4 electrode device. This sensor is able to determine soil resistivity distribution from a determined soil volume and was used to investigate soil properties at a depth of 1 m (Panissod et al. 1997 [9]). Measurements were made manually on sampling sites where final yield information was available according to the 15m × 9m grid. Values represent the average of five repetitions.

### 4.5 Vegetation index

For Field 1, commercial service Oenoview (ICV, Lattes, France) was used to provide maps of the FCover (Fraction of vegetation Cover) at 2.25 m² pixels (Rousseau et al. 2013 [10]; Tondriaux et al. 2018 [11]) derived from SPOT 6-7 satellite images. Images were acquired in early July 2022. The FCover is similar to GLCV (Green Leaf CoVer area).

For Field 2, NDVI (Normalized Difference Vegetation Index) was computed from a multi-spectral airborne image, with 1 m resolution as described in Acevedo-Opazo et al. [2]. The image was acquired in August 2007 by “L'avion Jaune” at a 3200 m elevation under clear sky and dry soil conditions. The images were first geo-referenced using relevant points on the image such as field corners or obvious end of row. The co-ordinates of these features were determined using a DGPS (Differential Global Positioning System) (Leica Geosystems company, model GS 50 with differential correction OMNISTAR). NDVI was computed pixel-by-pixel from two spectral regions contained in the images (i) red (632–695 nm) and (ii) near-infrared (757–853 nm). To avoid the effect of canopy cover discontinuity due to the vine training system (simple trellis), an averaged NDVI calculation was made using a 3 × 3 pixel-moving average window (area of 9 m²) around yield sampling sites.

### 4.6 Aggregated data

For each field, aggregated dataset was generated using QGIS (QGIS Association, Switzerland). A more regular map of vine location was computed from rows anchors location and vine spacing. Yield component data were then attributed to each vine of this map based on their location.

## Ethics Statement

The authors declare that there are no ethical issues with the data presented.

## CRediT authorship contribution statement

**Baptiste Oger:** Conceptualization, Methodology, Investigation, Writing – original draft. **Yulin Zhang:** Investigation, Writing – review & editing. **Jean-Philippe Gras:** Investigation, Writing – review & editing. **Yoann Valloo:** Investigation, Writing – review & editing. **Pauline Faure:** Investigation. **Guilhem Brunel:** Writing – review & editing. **Bruno Tisseyre:** Conceptualization, Writing – review & editing, Supervision.

## Data Availability

High spatial resolution dataset of grapevine yield components at the within-field level (Original data) (zenodo).
